# Causal associations between autoimmune diseases and cognitive impairment: A Mendelian randomization study

**DOI:** 10.1097/MD.0000000000046835

**Published:** 2026-01-02

**Authors:** Xue Jin, Jing Wang, Yu-Jie Du, Yi-Yuan Wang, Zhang-Wei Lu, Bao-Zhu Li

**Affiliations:** a Department of Rheumatology and Immunology, Second Affiliated Hospital of Anhui Medical University, Hefei, China; b Center for Big Data and Population Health of IHM, Department of Epidemiology and Biostatistics, School of Public Health, Anhui Medical University, Hefei, China; c Anhui Provincial Laboratory of Inflammatory and Immune Diseases, Hefei, China.

**Keywords:** ankylosing spondylitis, celiac disease, cognitive impairment, Mendelian randomization study, systemic lupus erythematosus, type 1 diabetes

## Abstract

Epidemiological evidence suggests that there is an association between systemic lupus erythematosus (SLE), type 1 diabetes (T1D), ankylosing spondylitis (AS), celiac disease (CD), and cognitive impairment (CI). However, the causal relationship between SLE, T1D, AS, CD, and CI remains unclear. The causal effects of the instrumental variables were analyzed using the random-effects inverse variance weighted method. Horizontal pleiotropy was examined by sensitivity analyses applying the weighted median method and the Mendelian randomization-Egger method. In order to avoid bias resulting from single-nucleotide polymorphisms, a leave-one-out analysis was employed. Our Mendelian randomization study identified the causality between SLE, CD, and declining cognitive performance (OR = 1.01, 95% CI: 1.001–1.030, *P* = .04; OR = 1.02, 95% CI: 1.02–1.04, *P* = .01), and the causality between CD and cognitive function (OR = 1.03, 95% CI: 1.01–1.05, *P* = .01). There was no evidence of a causative link between T1D, AS, and cognitive performance (OR = 1.00, 95% CI: 1.00–1.01, *P* = .23; OR = 1.01, 95% CI: 0.98–1.05, *P* = .46). And no causal relationship was found between SLE, T1D, AS and cognitive function (OR = 0.99, 95% CI: 0.96–1.02, *P* = .38; OR = 1.01, 95% CI: 0.97–1.05, *P* = .59; OR = 1.01, 95% CI: 0.98–1.01, *P* = .81). Our findings support causal relationships between SLE, CD, and declining cognitive performance, and between CD and cognitive function in European populations. No causal association was discovered between T1D, AS, and declining cognitive performance, or between SLE, T1D, AS and cognitive function.

## 1. Introduction

A prevalent chronic condition linked to aging is cognitive impairment (CI), which includes deterioration of cognitive function and performance.^[1]^ Problems with focus, memory loss, multitasking, and problem-solving are all signatures of CI. An increasing body of evidence indicates that individuals with autoimmune diseases, including systemic lupus erythematosus (SLE), ankylosing spondylitis (AS), type 1 diabetes (T1D), rheumatoid arthritis (RA), and celiac disease (CD), may additionally have CI. This could be relevant to people with autoimmune disorders who use anti-inflammatory medications often or for an extended period of time, which lowers the volume of the hippocampus and interferes with hippocampal function.^[2–4]^

One of the most frequent neuropsychiatric symptoms of SLE, characterized as “brain fog,” is mild CI.^[5,6]^ A cohort study found that “brain fog” is common in SLE patients and may raise the chance of depression or nervousness in SLE patients.^[7]^ However, a prospective study conducted in Russia suggested that the occurrence of CI in T1D patients is related to the dysregulation of glucose metabolism, including the impairment of psychomotor function caused by high blood sugar and the increase of oxidative stress caused by low blood sugar.^[8]^ This dysregulation increases the risk of dementia by damaging neurons.^[9]^ Through prospective follow-up, a study discovered that the mechanism of cognitive complications in CD may be related to the intake of gluten, causing complications and leading to brain damage.^[10]^ Besides, bipolar disorder, schizophrenia, and other mental disorders may be linked to CD.^[11]^ According to a cohort study conducted in Brazil, individuals with AS, who are at risk of cognitive impairment, exhibited altered neural networks and regional brain activity. They also often had neuropsychiatric symptoms.^[12]^

Evidence of a causal connection between RA and an elevated risk of cognitive performance and cognitive function has been identified in a recent Mendelian randomization (MR) study.^[13]^ And common autoimmune disorders feature a similar pathophysiological mechanism and genetic predisposition. Nevertheless, the causal relationship between CI and other autoimmune disorders remains unclear. Therefore, we used MR analysis to clarify the causal links between SLE, T1D, AS, CD, and CI.

## 2. Methods

### 2.1. The assumptions of MR and instrumental selection

**Figure 1.**
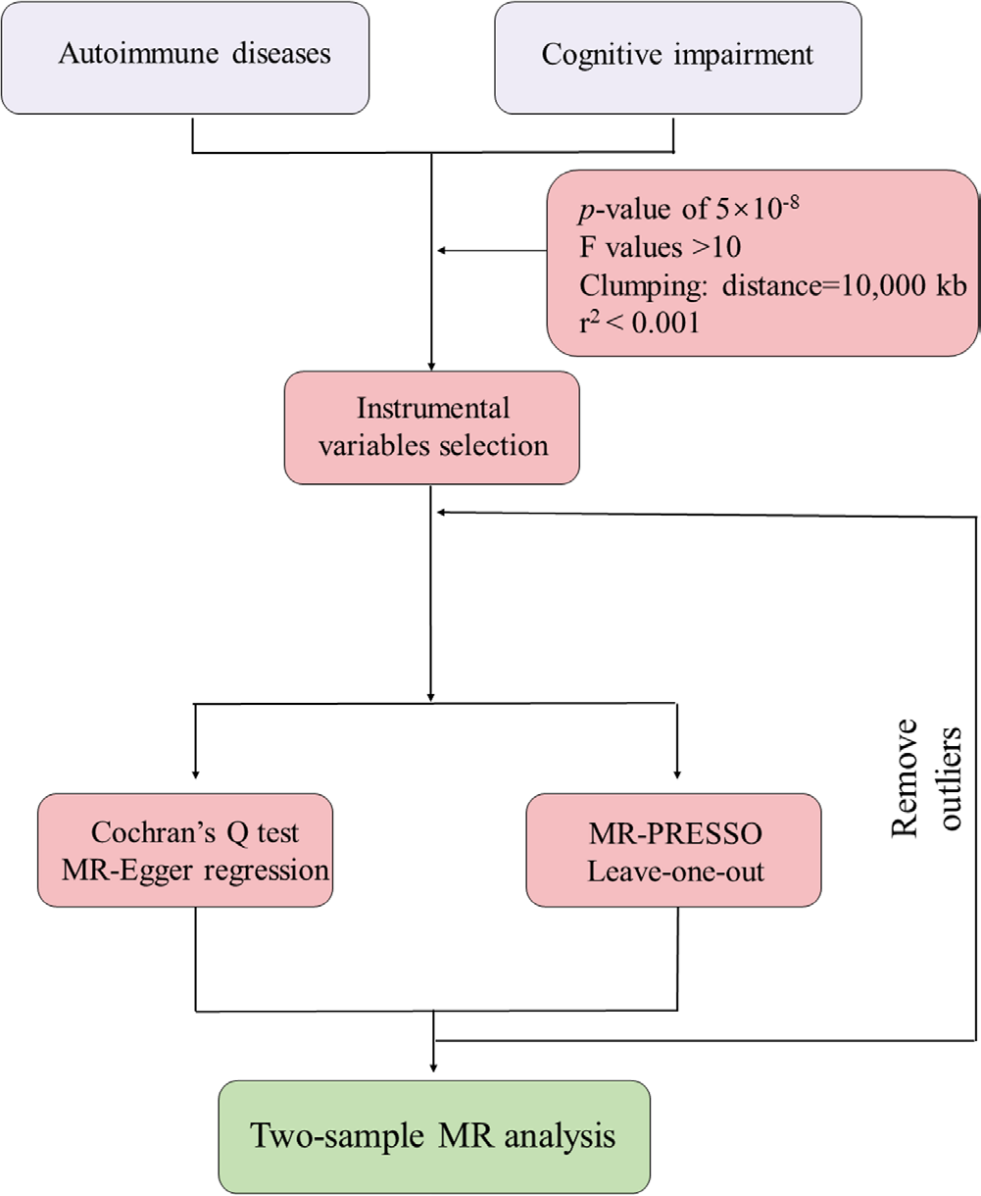
Flowchart of this MR study. MR = Mendelian randomization, MR‐PRESSO = MR‐pleiotropy residual sum and outlier.

The two-sample MR package in R (version 4.2.3; University of Bristol, Bristol, UK) was used. The *F* and *r*^2^ values were calculated to ensure each single-nucleotide polymorphism (SNP)’s validity. SNPs significantly associated with exposure (*P* < 5 × 10^−8^) were chosen as instrumental variables. Linkage disequilibrium was eliminated in our study (*r*^2^ < 0.001, 10,000 kb). Palindromic SNPs with intermediate allele frequencies and SNPs with *F*-values < 10 were removed. Detailed information on the SNPs significantly associated with our exposures was shown in Tables S2 and S3, Supplemental Digital Content, https://links.lww.com/MD/R41. Ethical approval was unnecessary, as the reanalysis of aggregated summary-level data from previous studies. Figure S1, Supplemental Digital Content, https://links.lww.com/MD/R41 illustrates our study’s assumptions and design. The 3 main assumptions on which the MR analysis is based are presented in Table S1, Supplemental Digital Content, https://links.lww.com/MD/R41. In Figure 1, the flowchart of our study design is shown.

### 2.2. Data sources

Exposures fall into 4 categories, and the genome-wide association study (GWAS) data can be obtained from the IEU Open GWAS database (https://gwas.mrcieu.ac.uk/datasets/) and the European Bioinformatics Institute GWAS Catalogue (https://www.ebi.ac.uk/). The exposures we selected include systemic lupus erythematosus (ID: ebi-a-GCST90018917), type 1 diabetes (ID: ebi-a-GCST90018925), ankylosing spondylitis (ID: ebi-a-GCST005529), and celiac disease (ID: ieu-a-1058).

The outcomes’ GWAS data were also from the IEU Open GWAS database and the GWAS catalogue. The results considered for analysis were as follows: cognitive performance (ID: ebi-a-GCST006572) and cognitive function (ID: ieu-b-4838). Racial disparities were not present, since every sample was of European ancestry. Table 1 shows the summary statistics for exposure and outcome. As replication samples, we also employed additional SLE, T1D, AS, and CD datasets to confirm the accuracy of our results. The GWAS data used in this study were ethically approved before citation.

**Table 1:** Descriptive details of the source of autoimmune diseases and cognitive impairment.

Trait	Population	Cases	Controls	Sample size	Data sources
Systemic lupus erythematosus	European	647	482,264	482,911	IEU OPEN GWAS
Systemic lupus erythematosus*	European	236	376,254	376,490	FinnGen
Type 1 diabetes	European	18,942	501,638	520,580	IEU OPEN GWAS
Type 1 diabetes*	European	8967	308,373	317,340	FinnGen
Ankylosing spondylitis	European	9069	13,578	22,647	IEU OPEN GWAS
Ankylosing spondylitis*	European	1193	374,621	375,814	FinnGen
Celiac disease	European	11,812	11,837	23,649	IEU OPEN GWAS
Celiac disease*	European	4533	10,750	15,283	IEU OPEN GWAS
Cognitive performance	European	–	–	257,841	IEU OPEN GWAS
Cognitive function	European	–	–	22,593	IEU OPEN GWAS

The 2 sets of SNPs associated with these autoimmune diseases are independent.

GWAS = genome-wide association study, IEU = integrative epidemiology unit.

* Summary data of 4 autoimmune diseases from GWAS as a verification.

### 2.3. Statistical methods

The MR-Egger method, weighted median (WM), and inverse variance weighted (IVW) method were the main methods employed. And the IVW approach served as our primary evaluation tool. Also, there was no relation detected between the selected SNPs and alcohol or tobacco use, which might potentially exist as confounding variables for autoimmune illnesses such as SLE, T1D, AS, and CD.

### 2.4. Sensitivity analysis

Sensitivity analyses using the Cochran *Q* test, MR-PRESSO, MR-Egger regression, and leave-one-out analysis have been carried out to validate the accuracy of the results we obtained. The SNPs heterogeneity was examined using Cochran *Q* test.^[14]^ Pleiotropy is used to describe genetic variability that affects outcomes through several paths.^[15]^ Pleiotropy at the gene level was explored using MR-PRESSO. It finds variations with pleiotropic effects and reflects the impact of genetic variants on causal estimates. An additional method for assessing the existence of pleiotropy was MR-Egger regression. An intercept with statistical significance can be used to identify pleiotropy. It obtains the linear regression of the genetic variations on the outcome. To assess the overall influence of certain genetic variations, a leave-one-out analysis was used.^[16]^

## 3. Results

### 3.1. MR analysis of autoimmune diseases on cognitive performance

**Figure 2.**
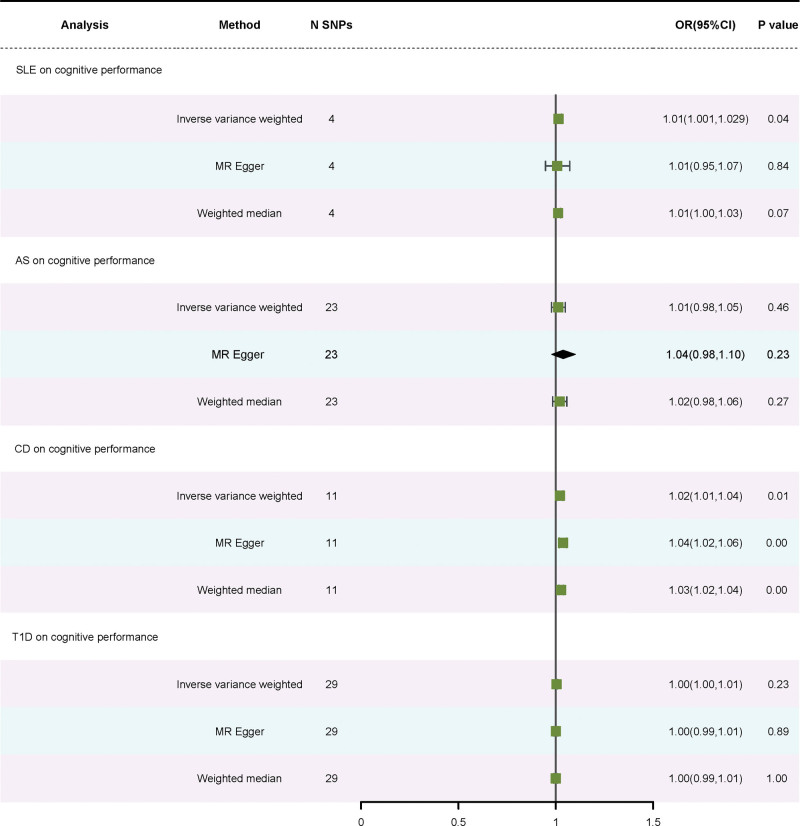
Forest plot of SNPs associated with 4 autoimmune diseases on declining cognitive performance. SNPs = single-nucleotide polymorphisms.

Using the IVW method, a link of causation between SLE and cognitive performance was discovered (OR = 1.01, 95% CI: 1.001–1.030, *P* = .04). The WM approach and the MR-Egger approach failed to find this association. Significant heterogeneity existed (*P* = .03). Our findings are deemed credible given no indication of horizontal pleiotropy (*P* = .84). Using the IVW approach, a statistically significant correlation between CD and cognitive performance was discovered (OR = 1.02, 95% CI: 1.01–1.04, *P* = .01). This correlation was also identified by applying the WM method and the MR-Egger approach. Significant heterogeneity (*P* = .00) existed. There was no horizontal pleiotropy (*P* = .05). No causal link between T1D, AS, and cognitive performance was found using the IVW method (OR = 1.00, 95% CI: 1.00–1.01, *P* = .23; OR = 1.01, 95% CI: 0.98–1.05, *P* = .46). Significant heterogeneity existed (*P* = .04; *P* = .01). There was no horizontal pleiotropy (*P* = .60; *P* = .32). Figure S2, Supplemental Digital Content, https://links.lww.com/MD/R41 presents the funnel plot, whereas Figure S3, Supplemental Digital Content, https://links.lww.com/MD/R41 displays the scatter plot. The stability of the MR estimates was further validated through a leave-out test (Figure S4, Supplemental Digital Content, https://links.lww.com/MD/R41). Table 2 lists the statistical findings of various autoimmune diseases on cognitive performance. In Figure 2, the forest plot is displayed.

**Table 2:** Mendelian randomization estimates of different autoimmune diseases on cognitive performance.

Exposure	Method	OR	95% CI	*P*-value	MR-Egger intercept (*P*-value)
SLE	IVW	1.01	1.001–1.029	**.04**	0.00 (.84)
	MR-Egger	1.01	0.95–1.07	.84	
	WM	1.01	1.00–1.02	.07	
SLE*	IVW	1.01	1.001–1.030	**.02**	
	MR-Egger	1.00	0.95–1.07	.27	‐0.01 (.73)
	WM	1.01	1.00–1.03	**.00**	
T1D	IVW	1.00	0.99–1.01	.53	0.00 (.60)
	MR-Egger	1.00	0.97–1.01	.45	
	WM	0.99	0.97–1.01	.60	
T1D*	IVW	1.00	1.00–1.01	.23	
	MR-Egger	1.00	0.99–1.01	.89	0.00 (.45)
	WM	1.00	0.99–1.01	1.00	
AS	IVW	1.01	0.98–1.05	.46	0.00 (.32)
	MR-Egger	1.04	0.98–1.10	.23	
	WM	1.02	0.98–1.06	.27	
AS*	IVW	1.00	0.99–1.01	.41	
	MR-Egger	1.01	1.00–1.02	.10	‐0.01 (.12)
	WM	1.00	0.99–1.00	.74	
CD	IVW	1.02	1.01–1.04	**.01**	‐0.01 (.05)
	MR-Egger	1.04	1.02–1.06	**.00**	
	WM	1.03	1.02–1.04	**1.04E-06**	
CD*	IVW	1.01	1.01–1.02	**1.99E-07**	‐0.01 (.55)
	MR-Egger	1.04	1.02–1.05	**.00**	
	WM	1.01	1.00–1.04	**.00**	

*P* values in bold indicate statistical significance at *P* < .05. AS = ankylosing spondylitis, CD = celiac disease, CI = confidence interval, IVW = inverse variance weighted, OR = odds ratios, SLE = systemic lupus erythematosus, T1D = type 1 diabetes, WM = weighted median.

* Summary data of 4 autoimmune diseases from GWAS as a verification.

### 3.2. MR analysis of autoimmune diseases on cognitive function

**Figure 3.**
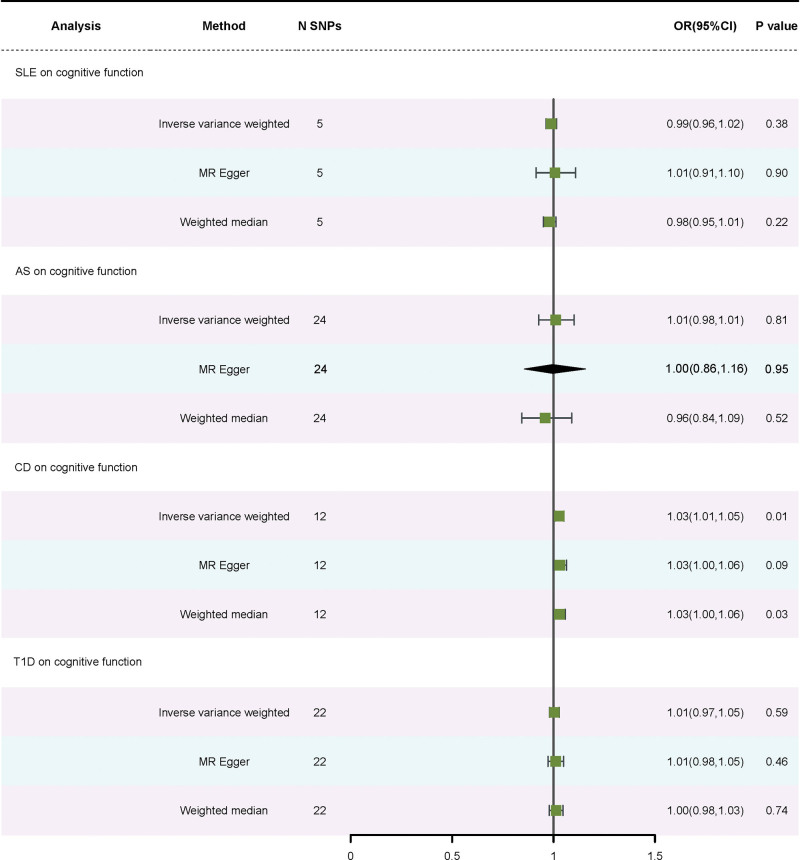
Forest plot of SNPs associated with 4 autoimmune diseases on declining cognitive function. SNPs = single-nucleotide polymorphisms.

Using the IVW approach, a correlation was found between CD and cognitive function (OR = 1.03, 95% CI: 1.01–1.05, *P *= .01). This correlation was also detected using the WM method (OR = 1.03, *P* = .03). There was no heterogeneity or horizontal pleiotropy (*P* = .61; *P* = .88). No causal association was discovered in the results of SLE, T1D, and AS on cognitive function using the IVW method (OR = 0.99, 95% CI: 0.96–1.02, *P* = .38; OR = 1.01, 95% CI: 0.97–1.05, *P* = .59; OR = 1.01, 95% CI: 0.98–1.01, *P* = .81). Significant heterogeneity (*P* = .86; *P *= 1.00; *P* = .32) did not exist. There was no horizontal pleiotropy (*P* = .72; *P *= .23; *P* = .82). Figure S5, Supplemental Digital Content, https://links.lww.com/MD/R41 provides the funnel plot, while Figure S6, Supplemental Digital Content, https://links.lww.com/MD/R41 lists the scatter plot. The stability of the MR estimates was further validated through a leave-out test (Figure S7, Supplemental Digital Content, https://links.lww.com/MD/R41). The statistical results of different autoimmune diseases on cognitive function are summarized in Table 3. The forest plot is displayed in Figure 3.

**Table 3:** Mendelian randomization estimates of different autoimmune diseases on cognitive function.

Exposure	Method	OR	95% CI	*P*-value	MR-Egger intercept (*P*-value)
SLE	IVW	0.99	0.96–1.02	.38	‐0.01 (.72)
	MR-Egger	1.01	0.91–1.10	.90	
	WM	0.98	0.95–1.01	.22	
SLE*	IVW	1.02	0.99–1.10	.19	
	MR-Egger	1.01	0.93–1.10	.85	0.01 (.82)
	WM	1.02	0.99–1.05	.22	
T1D	IVW	0.97	0.94–1.00	.05	0.01 (.23)
	MR-Egger	0.94	0.89–1.00	.05	
	WM	0.95	0.92–1.00	.02	
T1D*	IVW	1.00	0.98–1.03	.74	
	MR-Egger	1.01	0.97–1.05	.59	0.00 (.68)
	WM	1.01	0.98–1.05	.46	
AS	IVW	1.01	0.93–1.01	.81	0.00 (.82)
	MR-Egger	1.00	0.86–1.16	.95	
	WM	0.96	0.84–1.09	.52	
AS*	IVW	0.99	0.97–1.01	.23	
	MR-Egger	0.97	0.95–1.00	.09	0.01 (.15)
	WM	0.98	0.97–1.00	0.10	
CD	IVW	1.00	0.99–1.02	.66	0.00 (.49)
	MR-Egger	1.00	0.99–1.03	.42	
	WM	1.01	0.98–1.02	.89	
CD*	IVW	1.02	1.00–1.05	.10	
	MR-Egger	0.99	0.89–1.09	.80	0.01 (.49)
	WM	1.02	1.00–1.05	.30	

AS = ankylosing spondylitis, CD = celiac disease, CI = confidence interval, IVW = inverse variance weighted, OR = odds ratios, SLE = systemic lupus erythematosus, T1D = type 1 diabetes, WM = weighted median.

* Summary data of 4 autoimmune diseases from GWAS as a verification.

## 4. Discussion

This is the first study that we are aware of that looks at the causal link between multiple autoimmune diseases and CI using MR analysis. Our findings point to causal associations between CD, SLE, and cognitive performance.

Prednisolone usage in SLE patients has been observed to be closely linked to “brain fog,” which is a condition that frequently results from central and peripheral nervous system dysfunction triggered by SLE.^[17,18]^ Additionally, there was a relationship identified between higher levels of serum pro-inflammatory cytokines and certain autoimmune processes that target NMDA receptors and cognitive disorders. In patients with SLE, memory impairment caused by autoantibodies against N-methyl-aspartate receptors or vascular lesions may result in hippocampus shrinkage and neuron loss.^[10,19]^ Poor lipid metabolism, along with an imbalance in soluble amyloid-β plasma and disruption of the neurovascular network, are the main causes of cognitive issues in T1D patients.^[20,21]^ Dementia and CI are more likely when brain glucose transport and insulin signaling mechanisms are compromised. For instance, a study has shown that the brains of dementia patients contain significantly fewer insulin-dependent glucose transporters (GLUT1, GLUT3).^[22,23]^ Similarly, in the hippocampus of rats, deletion of insulin receptors and insulin-like growth factor-1 receptors negatively impacts brain signaling pathways, ultimately leading to a loss in cognitive function.^[24]^ In genetically predisposed individuals, gluten intake causes CD; recently, mechanisms explaining the adverse effects of gluten-related disease on cognitive function have been postulated.^[10]^ In CD patients, increased levels of circulating cytokines are linked to behavioral, emotional, and cognitive abnormalities. High concentrations of pro-inflammatory cytokines in the bloodstream attach to the blood–brain barrier and promote the migration of white blood cells to the brain.^[25]^ White blood cells exacerbate inflammation in the brain by slowing down neuronal transmission, which impairs cognitive function.^[26]^ Additionally, it has been determined that AS and other systemic inflammatory disorders are risk factors for early cognitive decline.^[26,27]^ Due to their frequent use of glucocorticoid medication, these individuals are more likely to experience dementia, an elevated risk of cerebrovascular disease, and further brain damage.^[28–30]^ An extra possibility might be that those with AS have elevated blood amyloid protein levels, which impact everyday activities and disrupt sleep, ultimately leading to cognitive loss.^[31]^

Previous observational studies have used questionnaires to assess the cognitive status of patients, mostly in groups of <200. These studies have some limitations. Firstly, a lot of the studies had small sample sizes and were unable to find correlations with specific cognitive impairment subgroups. Secondly, it is challenging to completely rule out the chance of confounding variables, including physical activity, mood disorders, and poor sleep quality, that were not examined in the investigation. Thirdly, no repeat tests were conducted throughout the neuropsychological evaluation of the patients, and their stability was in doubt.

There are some limitations to our cause-and-effect study. First, the study’s population for exposure and outcomes was limited to Europeans, which is also a limitation of most studies. If the findings hold true for other populations, it is unknown. Besides, the GWAS data on various degrees of cognitive decline were not available to us, making us unable to evaluate the data in subgroups. For future analyses, larger and more comprehensive data sets may be needed, and generalizable to other, larger populations. In conclusion, we found a causal association between CD, SLE, and cognitive performance. No causal association was discovered between CD, SLE, and cognitive function, T1D, AS, and cognitive impairment.

## Acknowledgments

We appreciate all the volunteers who participated in this study. We are grateful to the Open GWAS for providing GWAS summary statistics.

## Author contributions

**Conceptualization:** Bao-Zhu Li.

**Data curation:** Xue Jin, Jing Wang.

**Formal analysis:** Yu-Jie Du.

**Methodology:** Yu-Jie Du, Yi-Yuan Wang, Zhang-Wei Lu.

**Project administration:** Xue Jin, Jing Wang.

**Resources:** Bao-Zhu Li.

**Software:** Yu-Jie Du.

**Supervision:** Bao-Zhu Li.

**Validation:** Yi-Yuan Wang, Zhang-Wei Lu.

**Visualization:** Xue Jin, Jing Wang.

**Writing – original draft:** Xue Jin, Jing Wang.

**Writing – review & editing:** Bao-Zhu Li.

## Supplementary Material


