# Supplementary material for: Causal associations between autoimmune diseases and cognitive impairment: A Mendelian randomization study
Source: Medicine (Baltimore). 2026 Jan 2;105(1):e46835. doi: 10.1097/MD.0000000000046835 (PMC12778153; doi:10.1097/MD.0000000000046835)
Supplement: Supplementary file 1 [file medi-105-e46835-s001.pdf]

Fig. S1 Diagram of MR study design

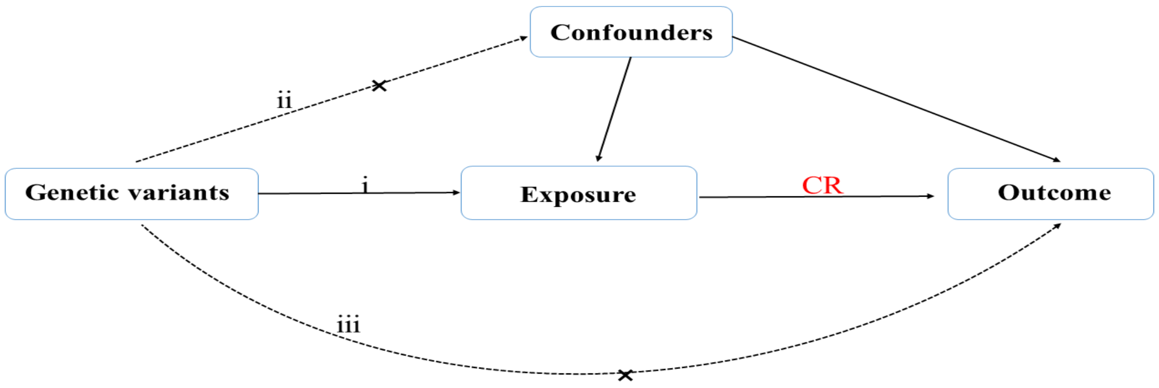

Fig. S2 Funnel plots for four autoimmune diseases on cognitive performance

(A) Funnel plot for SLE on cognitive performance

(B) Funnel plot for T1D on cognitive performance

(C) Funnel plot for AS on cognitive performance

(D) Funnel plot for CD on cognitive performance

MR, Mendelian randomization; SE, standard error.

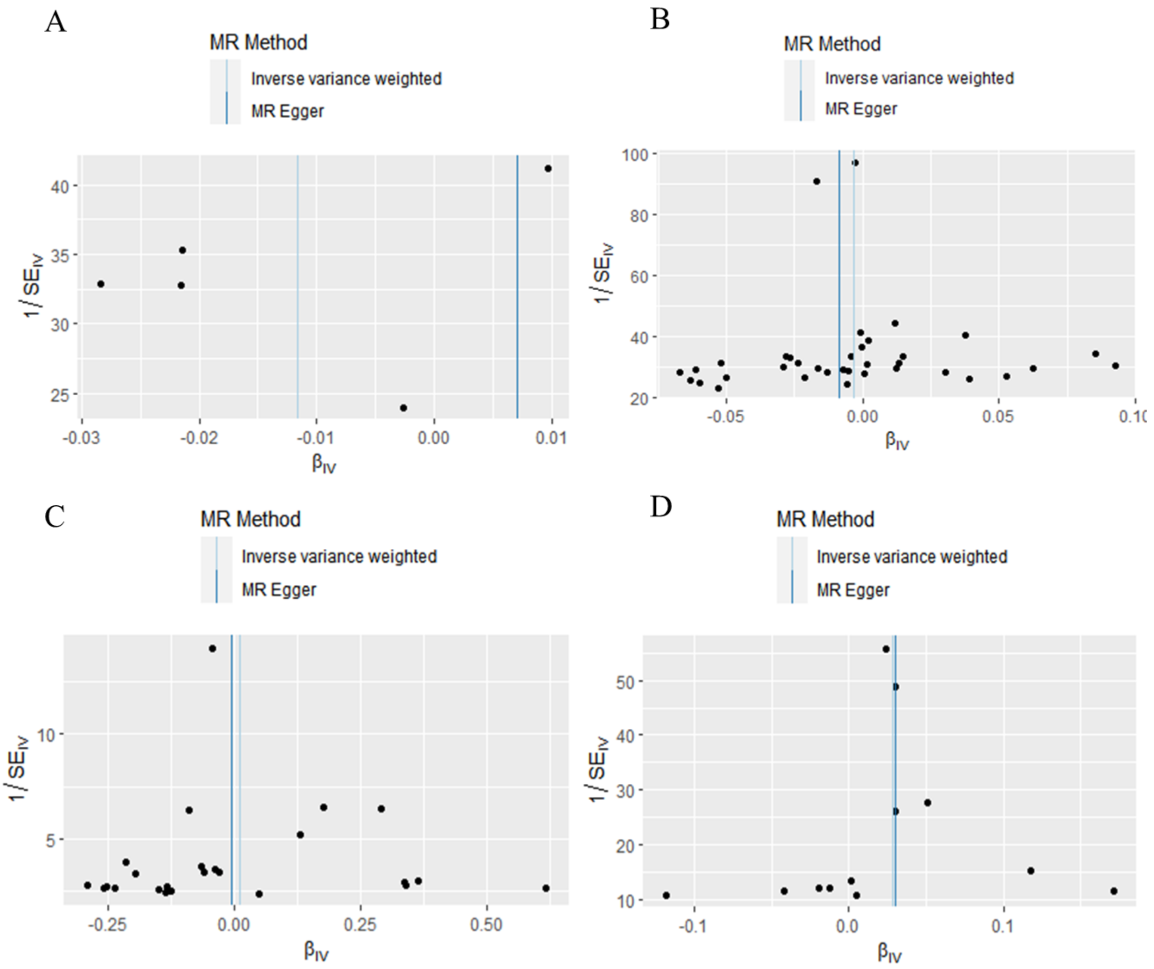

Fig. S3 Scatter plots for four autoimmune diseases on cognitive performance

(A) Scatter plot for SLE on cognitive performance

(B) Scatter plot for T1D on cognitive performance

(C) Scatter plot for AS on cognitive performance

(D) Scatter plot for CD on cognitive performance

MR, Mendelian randomization; SE, standard error.

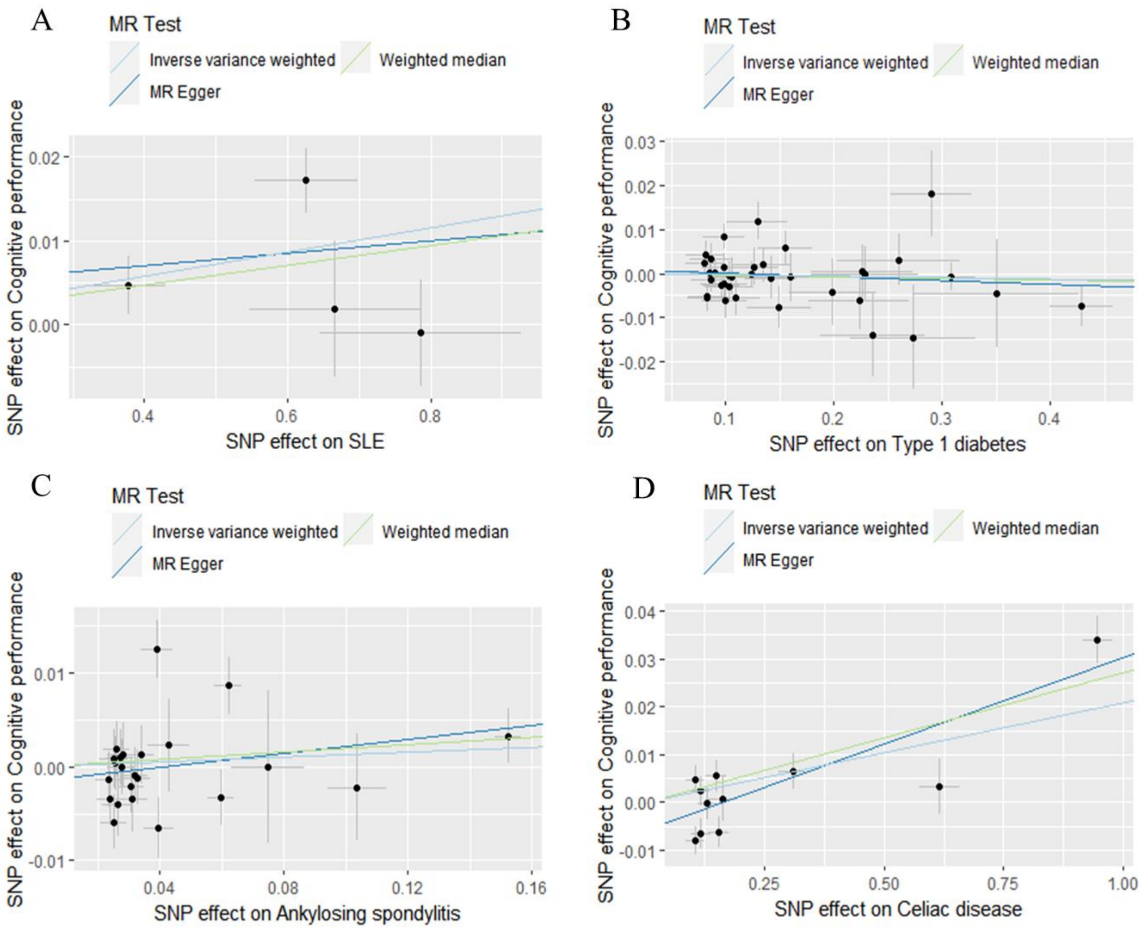

Fig. S4 Leave□one□out sensitivity analysis for four autoimmune diseases on cognitive performance

(A) Leave□one□out sensitivity analysis for SLE on cognitive performance

(B) Leave□one□out sensitivity analysis for T1D on cognitive performance

(C) Leave□one□out sensitivity analysis for AS on cognitive performance

(D) Leave□one□out sensitivity analysis for CD on cognitive performance

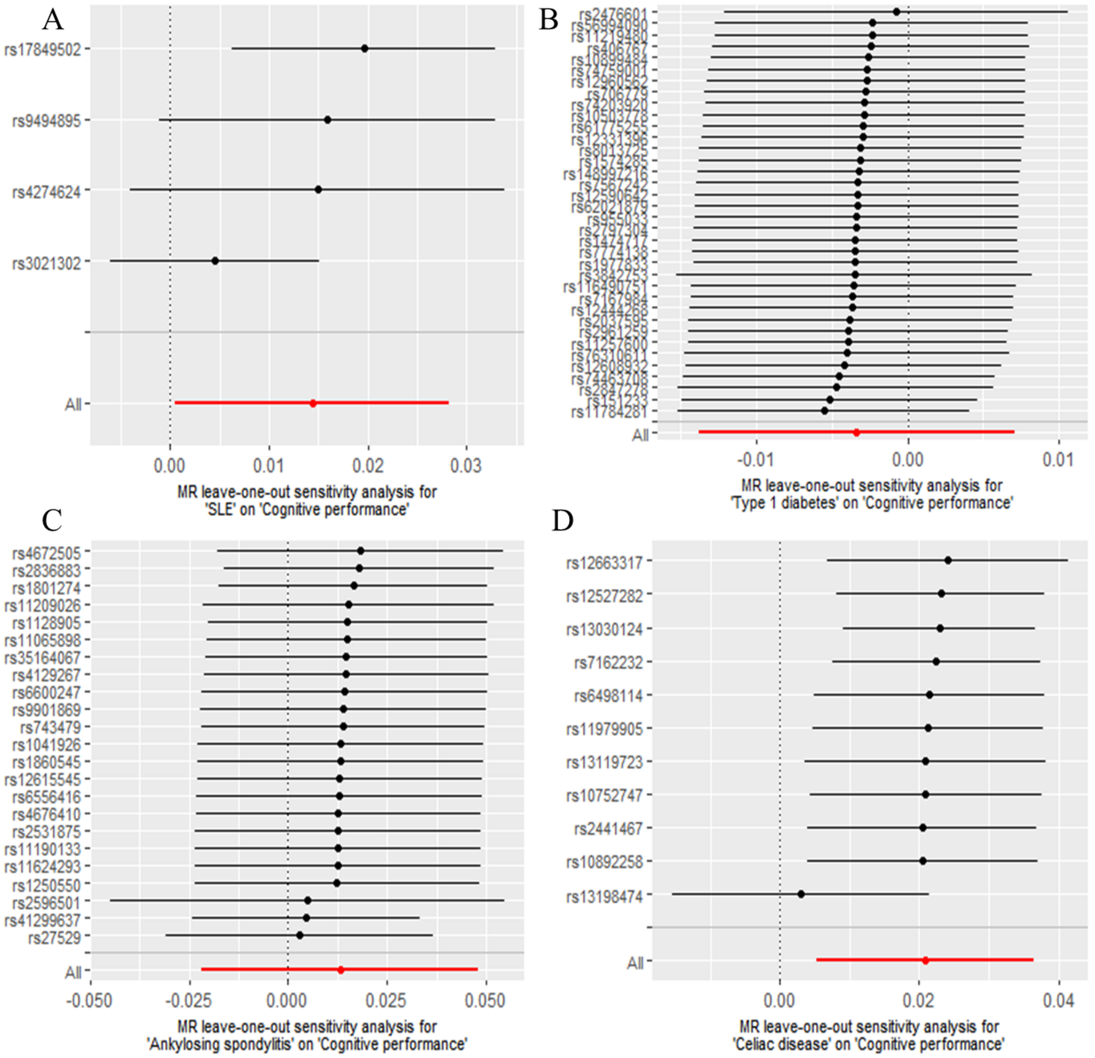

Fig. S5 Funnel plots for four autoimmune diseases on cognitive function

(A) Funnel plot for SLE on cognitive function

(B) Funnel plot for T1D on cognitive function

(C) Funnel plot for AS on cognitive function

(D) Funnel plot for CD on cognitive function

MR, Mendelian randomization; SE, standard error.

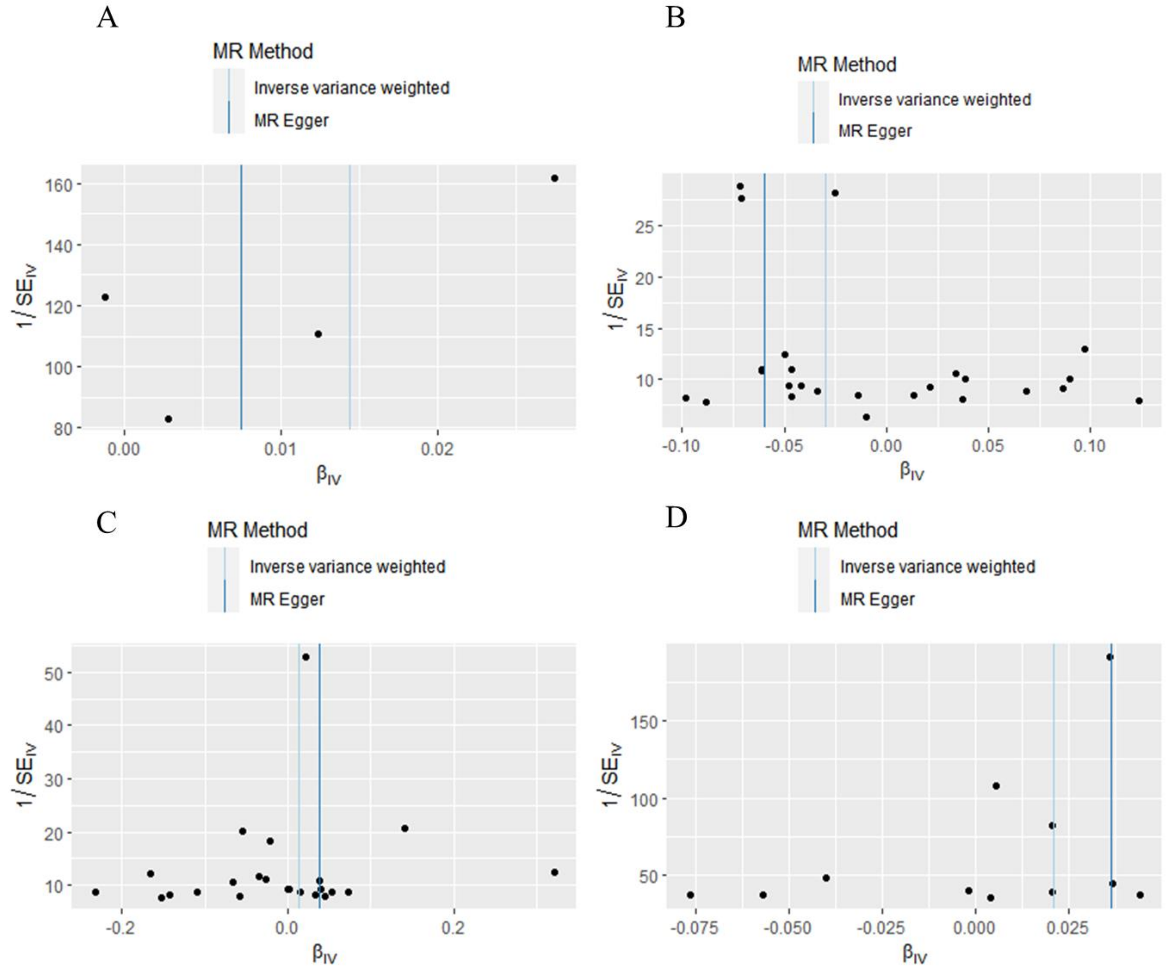

Fig. S6 Scatter plots for four autoimmune diseases on cognitive function

(A) Scatter plot for SLE on cognitive function

(B) Scatter plot for T1D on cognitive function

(C) Scatter plot for AS on cognitive function

(D) Scatter plot for CD on cognitive function

MR, Mendelian randomization; SE, standard error.

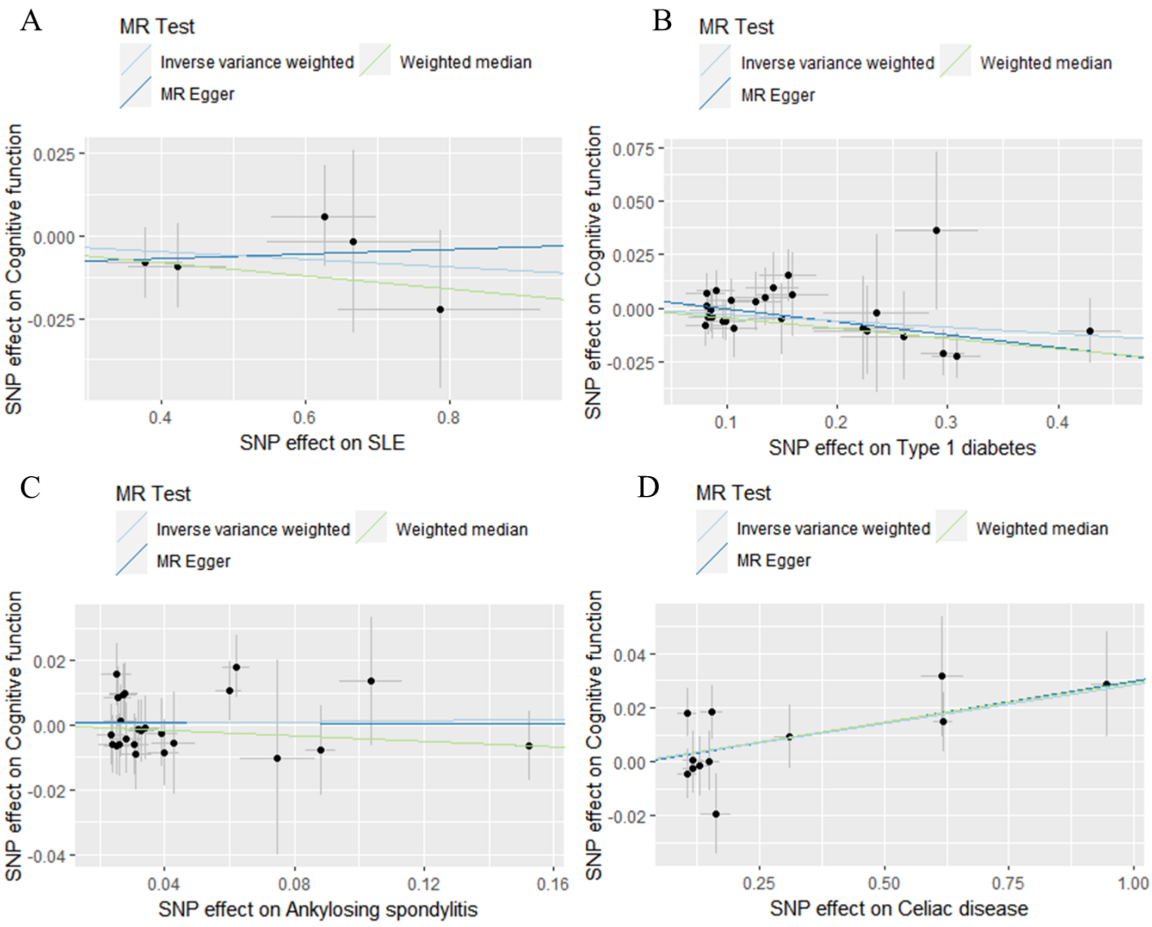

Fig. S7 Leave□one□out sensitivity analysis for four autoimmune diseases on cognitive function

(A) Leave□one□out sensitivity analysis for SLE on cognitive function

(B) Leave□one□out sensitivity analysis for T1D on cognitive function

(C) Leave□one□out sensitivity analysis for AS on cognitive function

(D) Leave□one□out sensitivity analysis for CD on cognitive function

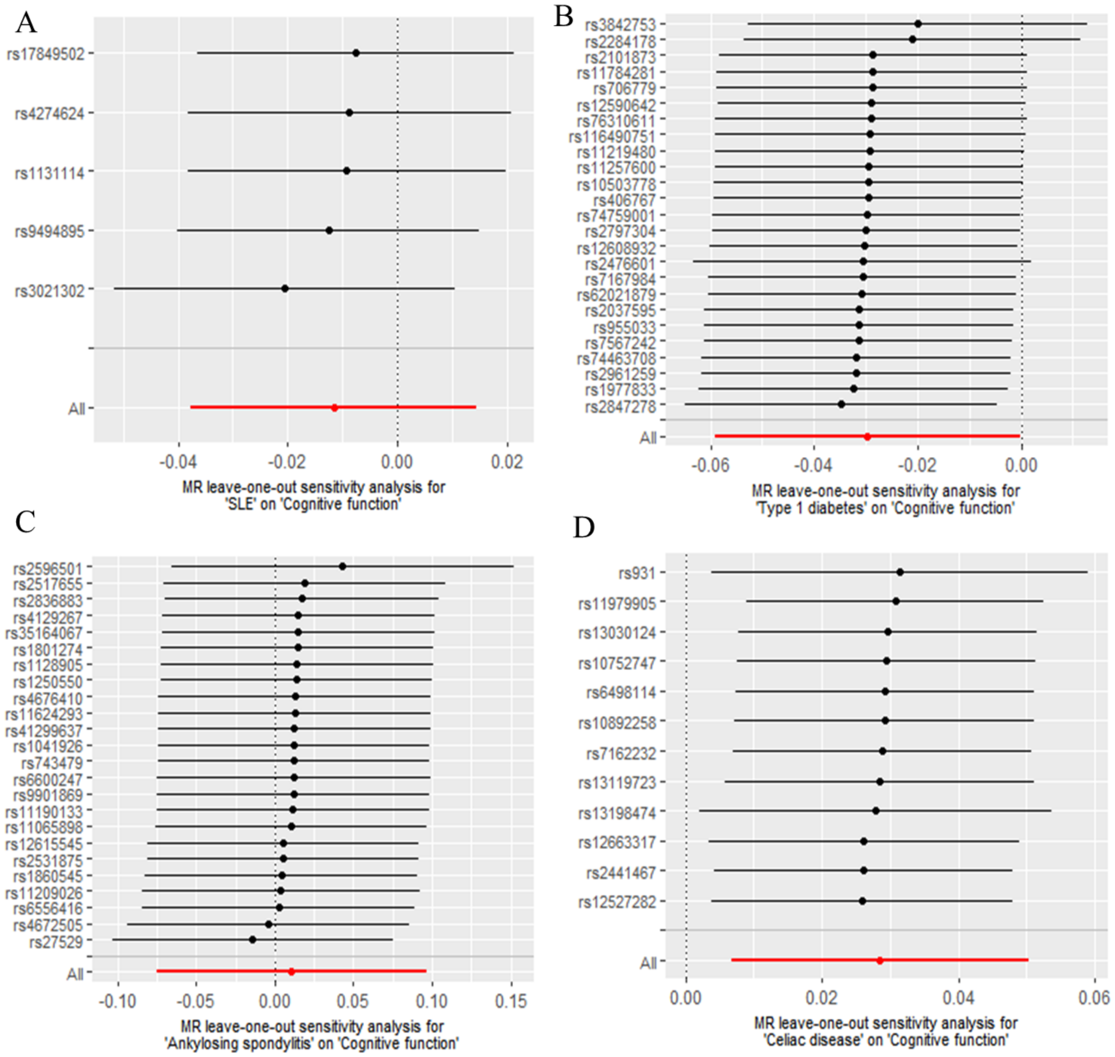

Supplementary Table S1 The main assumptions on the MR Analysis

**1**	Instrumental variables (IVs) are supposed to be strongly linked with exposures.
**2**	IVs should be independent from confounding factors associated with the relationship between the exposures and outcomes.
**3**	Relevance only through exposures to influence the results.

Supplementary Table S2 Characteristics of the genetic instrumental variables used in the two-sample MR analysis of autoimmune diseases on cognitive performance

Exposure	SNP	A1	A2	β	SE	*P*	N
SLE	rs17849502	T	G	0.79	0.14	1.95E-08	482911
SLE	rs3021302	C	T	0.63	0.07	5.25E-18	482911
SLE	rs4274624	T	C	-0.38	0.05	9.37E-13	482911
SLE	rs9494895	T	C	0.67	0.12	2.31E-08	482911
T1D	rs10503778	G	A	0.22	0.05	1.34E-06	457695
T1D	rs10899484	G	C	-0.10	0.02	2.78E-06	457695
T1D	rs11219480	C	A	-0.08	0.02	2.21E-06	457695
T1D	rs11257600	T	G	0.09	0.02	4.84E-06	457695
T1D	rs116490751	A	G	0.23	0.05	1.57E-06	457695
T1D	rs11784281	C	T	-0.10	0.02	1.71E-07	457695
T1D	rs12331396	T	G	-0.10	0.02	4.08E-06	457695
T1D	rs12444268	A	T	0.10	0.02	1.58E-06	457695
T1D	rs12590642	A	G	0.11	0.02	2.84E-06	457695
T1D	rs12608932	C	A	-0.08	0.02	4.54E-06	457695
T1D	rs12960562	T	C	-0.11	0.02	3.75E-06	457695
T1D	rs1474717	C	G	-0.23	0.05	3.41E-06	457695
T1D	rs148997216	A	G	-0.35	0.08	3.92E-06	457695
T1D	rs151233	T	C	0.13	0.03	4.05E-06	457695
T1D	rs1574285	T	G	-0.09	0.02	6.11E-07	457695
T1D	rs1977833	A	G	-0.09	0.02	2.25E-07	457695
T1D	rs2037595	G	A	0.13	0.03	7.27E-07	457695
T1D	rs2476601	G	A	-0.43	0.03	1.24E-50	457695
T1D	rs2797304	G	A	0.09	0.02	1.74E-06	457695
T1D	rs2847278	T	C	-0.16	0.03	1.42E-09	457695
T1D	rs2961259	A	G	-0.08	0.02	2.81E-06	457695
T1D	rs3842753	G	T	0.31	0.02	8.31E-44	457695
T1D	rs406767	C	T	0.15	0.03	4.73E-07	457695
T1D	rs56994090	C	T	-0.08	0.02	1.77E-06	457695
T1D	rs61775255	G	A	-0.10	0.02	4.14E-06	457695
T1D	rs62021879	A	G	-0.16	0.03	1.64E-06	457695
T1D	rs706779	C	T	-0.10	0.02	3.23E-08	457695
T1D	rs7167984	A	G	-0.13	0.02	2.75E-08	457695
T1D	rs74203920	T	C	0.27	0.06	2.27E-06	457695
T1D	rs74463708	A	G	-0.29	0.04	1.40E-14	457695
T1D	rs74759001	A	G	-0.24	0.05	9.99E-07	457695
T1D	rs7567242	A	C	0.14	0.02	4.17E-09	457695
T1D	rs76310611	A	C	-0.26	0.06	3.67E-06	457695
T1D	rs7774138	A	T	0.12	0.02	1.90E-09	457695
T1D	rs8013725	A	T	-0.20	0.04	1.01E-06	457695

Exposure	SNP	A1	A2	β	SE	*P*	N
T1D	rs955033	T	C	-0.10	0.02	1.49E-07	457695
AS	rs1041926	A	G	-0.07	0.01	1.55E-10	10619
AS	rs11065898	T	C	0.03	0.00	4.71E-08	10619
AS	rs11190133	T	C	-0.03	0.00	4.84E-14	10619
AS	rs11209026	A	G	-0.10	0.01	1.94E-27	10619
AS	rs1128905	C	T	-0.02	0.00	6.95E-09	10619
AS	rs11624293	C	T	0.04	0.01	1.49E-10	10619
AS	rs1250550	A	C	-0.03	0.00	1.46E-09	10619
AS	rs12615545	C	T	0.03	0.00	1.03E-09	10619
AS	rs1801274	G	A	0.03	0.00	1.35E-09	10619
AS	rs1860545	A	G	-0.03	0.00	2.78E-10	10619
AS	rs2531875	T	G	-0.03	0.00	1.22E-10	10619
AS	rs2596501	T	C	-0.15	0.00	1.00E-20	10619
AS	rs27529	G	A	-0.06	0.00	3.28E-47	10619
AS	rs2836883	A	G	-0.04	0.00	6.46E-17	10619
AS	rs35164067	A	G	-0.03	0.00	3.43E-10	10619
AS	rs4129267	T	C	-0.04	0.00	3.32E-13	10619
AS	rs41299637	G	T	-0.04	0.00	1.81E-15	10619
AS	rs4672505	G	A	-0.06	0.00	5.14E-47	10619
AS	rs4676410	A	G	0.03	0.00	9.90E-09	10619
AS	rs6556416	C	A	0.03	0.00	4.22E-08	10619
AS	rs6600247	C	T	0.03	0.00	2.58E-15	10619
AS	rs743479	T	C	-0.02	0.00	2.03E-08	10619
AS	rs9901869	A	G	0.03	0.00	6.04E-15	10619
CD	rs10752747	T	G	-0.12	0.02	5.05E-09	24265
CD	rs10892258	A	G	-0.15	0.02	1.73E-11	24263
CD	rs11979905	G	A	0.16	0.03	2.49E-08	24269
CD	rs12527282	T	C	-0.15	0.02	1.69E-13	24269
CD	rs12663317	C	A	-0.62	0.04	3.53E-47	24269
CD	rs13030124	A	G	-0.11	0.02	2.40E-08	24269
CD	rs13119723	G	A	-0.31	0.03	7.60E-29	24257
CD	rs13198474	A	G	0.95	0.03	1.00E-20	24262
CD	rs2441467	C	T	0.11	0.02	1.70E-08	24269
CD	rs6498114	T	G	-0.13	0.02	5.83E-10	24269
CD	rs7162232	A	G	-0.12	0.02	7.97E-09	24269

A1: effect_allele; A2: other_allele; *β*: beta; F: F□statistics; N: sample size; SE: standard error; SNP: single□nucleotide polymorphism; SLE: Systemic lupus erythematosus; T1D: Type 1 diabetes; AS: Ankylosing spondylitis; CD: Celiac disease.

Supplementary Table S3 Characteristics of the genetic instrumental variables used in the two-sample MR analysis of autoimmune diseases on cognitive function

Exposure	SNP	A1	A2	β	SE	*P*	N
SLE	rs1131114	C	T	0.42	0.07	4.36E-10	482911
SLE	rs17849502	T	G	0.79	0.14	1.95E-08	482911
SLE	rs3021302	C	T	0.63	0.07	5.25E-18	482911
SLE	rs4274624	T	C	-0.38	0.05	9.37E-13	482911
SLE	rs9494895	T	C	0.67	0.12	2.31E-08	482911
T1D	rs10503778	G	A	0.22	0.05	1.34E-06	457695
T1D	rs11219480	C	A	-0.08	0.02	2.21E-06	457695
T1D	rs11257600	T	G	0.09	0.02	4.84E-06	457695
T1D	rs116490751	A	G	0.23	0.05	1.57E-06	457695
T1D	rs11784281	C	T	-0.10	0.02	1.71E-07	457695
T1D	rs12331396	T	G	-0.10	0.02	4.08E-06	457695
T1D	rs12590642	A	G	0.11	0.02	2.84E-06	457695
T1D	rs12608932	C	A	-0.08	0.02	4.54E-06	457695
T1D	rs12960562	T	C	-0.11	0.02	3.75E-06	457695
T1D	rs151233	T	C	0.13	0.03	4.05E-06	457695
T1D	rs1574285	T	G	-0.09	0.02	6.11E-07	457695
T1D	rs1977833	A	G	-0.09	0.02	2.25E-07	457695
T1D	rs2037595	G	A	0.13	0.03	7.27E-07	457695
T1D	rs2476601	G	A	-0.43	0.03	1.24E-50	457695
T1D	rs2797304	G	A	0.09	0.02	1.74E-06	457695
T1D	rs2847278	T	C	-0.16	0.03	1.42E-09	457695
T1D	rs2961259	A	G	-0.08	0.02	2.81E-06	457695
T1D	rs3842753	G	T	0.31	0.02	8.31E-44	457695
T1D	rs406767	C	T	0.15	0.03	4.73E-07	457695
T1D	rs56994090	C	T	-0.08	0.02	1.77E-06	457695
T1D	rs61775255	G	A	-0.10	0.02	4.14E-06	457695
T1D	rs62021879	A	G	-0.16	0.03	1.64E-06	457695
T1D	rs706779	C	T	-0.10	0.02	3.23E-08	457695
T1D	rs7167984	A	G	-0.13	0.02	2.75E-08	457695
T1D	rs74463708	A	G	-0.29	0.04	1.40E-14	457695
T1D	rs74759001	A	G	-0.24	0.05	9.99E-07	457695
T1D	rs7567242	A	C	0.14	0.02	4.17E-09	457695
T1D	rs76310611	A	C	-0.26	0.06	3.67E-06	457695
T1D	rs955033	T	C	-0.10	0.02	1.49E-07	457695
AS	rs1041926	A	G	-0.07	0.01	1.55E-10	10619
AS	rs11065898	T	C	0.03	0.00	4.71E-08	10619
AS	rs11190133	T	C	-0.03	0.00	4.84E-14	10619
AS	rs11209026	A	G	-0.10	0.01	1.94E-27	10619
AS	rs1128905	C	T	-0.02	0.00	6.95E-09	10619
AS	rs11624293	C	T	0.049	0.01	1.49E-10	10619
AS	rs1250550	A	C	-0.03	0.00	1.46E-09	10619
AS	rs12615545	C	T	0.03	0.00	1.03E-09	10619
AS	rs1801274	G	A	0.03	0.00	1.35E-09	10619
AS	rs1860545	A	G	-0.034	0.00	2.78E-10	10619
AS	rs2517655	T	C	0.09	0.00	3.47E-80	10619

Exposure	SNP	A1	A2	β	SE	*P*	N
AS	rs2531875	T	G	-0.03	0.00	1.22E-10	10619
AS	rs2596501	T	C	-0.15	0.00	1.00E-20	10619
AS	rs27529	G	A	-0.06	0.00	3.28E-47	10619
AS	rs2836883	A	G	-0.04	0.00	6.46E-17	10619
AS	rs35164067	A	G	-0.03	0.00	3.43E-10	10619
AS	rs4129267	T	C	-0.03	0.00	3.32E-13	10619
AS	rs41299637	G	T	-0.04	0.00	1.81E-15	10619
AS	rs4672505	G	A	-0.06	0.00	5.14E-47	10619
AS	rs4676410	A	G	0.03	0.00	9.90E-09	10619
AS	rs6556416	C	A	0.03	0.00	4.22E-08	10619
AS	rs6600247	C	T	0.03	0.00	2.58E-15	10619
AS	rs743479	T	C	-0.02	0.00	2.03E-08	10619
AS	rs9901869	A	G	0.03	0.00	6.04E-15	10619
CD	rs10752747	T	G	-0.12	0.02	5.05E-09	24265
CD	rs10892258	A	G	-0.15	0.02	1.73E-11	24263
CD	rs11979905	G	A	0.16	0.03	2.49E-08	24269
CD	rs12527282	T	C	-0.15	0.02	1.69E-13	24269
CD	rs12663317	C	A	-0.62	0.04	3.53E-47	24269
CD	rs13030124	A	G	-0.11	0.02	2.40E-08	24269
CD	rs13119723	G	A	-0.31	0.03	7.60E-29	24257
CD	rs13198474	A	G	0.95	0.03	1.00E-200	24262
CD	rs2441467	C	T	0.11	0.02	1.70E-08	24269
CD	rs6498114	T	G	-0.13	0.02	5.83E-10	24269
CD	rs7162232	A	G	-0.12	0.02	7.97E-09	24269
CD	rs931	A	G	0.62	0.02	1.00E-20	24269

A1: effect_allele; A2: other_allele; *β*: beta; F: F□statistics; N: sample size; SE: standard error; SNP: single□nucleotide polymorphism; SLE: Systemic lupus erythematosus; T1D: Type 1 diabetes; AS: Ankylosing spondylitis; CD: Celiac disease.
